# Supplementary material for: Multiple domains of scaffold Tudor protein play non-redundant roles in Drosophila germline
Source: bioRxiv. 2025 Mar 17:2025.03.13.643173. Preprint. [Version 2] doi: 10.1101/2025.03.13.643173 (PMC11956945; doi:10.1101/2025.03.13.643173)
Supplement: Supplement 1 [file media-1.pdf]

Supplementary information

Multiple domains of scaffold Tudor protein play non-redundant roles in *Drosophila* germline

Samuel J. Tindell[1], Alyssa G. Boeving[1], Julia Aebersold[2] and Alexey L. Arkov[1]*

[1]Department of Biological Sciences, Murray State University, Murray, KY, USA
[2]Micro/Nano Technology Center, University of Louisville, Louisville, KY, USA

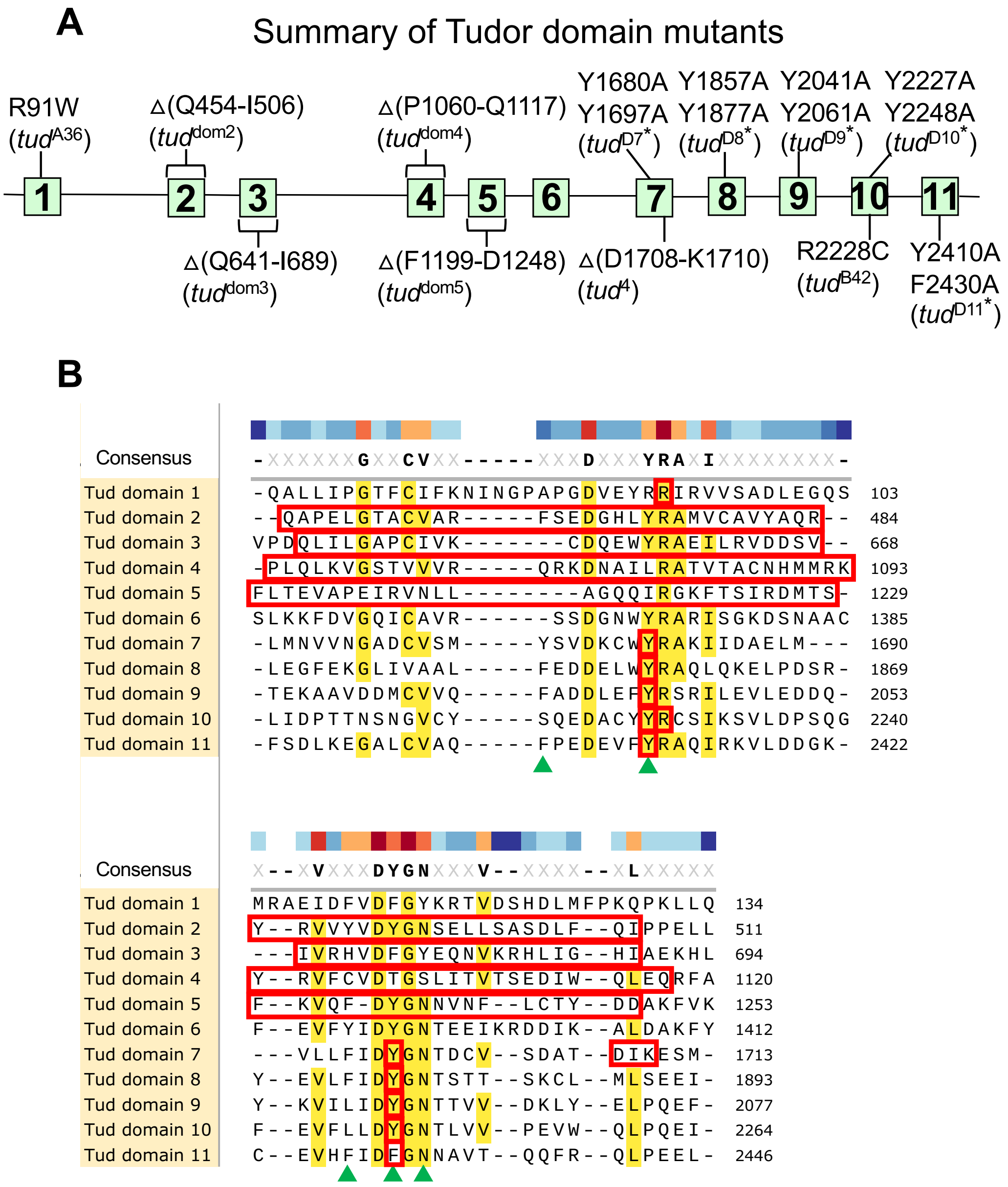

Figure S1. Summary of mutations in Tudor domains of Tudor protein.
(A) A diagram of Tud protein with all known Tud domains' mutations indicated in domains 1 (Arkov AL et al, 2006), 2-5 (this study) and domains 7-11 (Arkov AL et al, 2006, Boswell RE & Mahowald AP, 1985, Liu H et al, 2010). Deletion mutations are indicated as '∆'. Contrary to domains 2-5, CRISPR approach used in this study, failed to generate a comparable deletion of Tud domain 6 and also, previous mutational approaches did not isolate a mutation in this Tud domain. **(B)** Alignment of Tud domains shows the residues mutated in all Tud domain mutants shown in **(A)** (indicated in red). Part of this Figure, which shows the alignment, deletion mutations of Tud domains 2-5 and residues of methylarginine-binding pocket of Tud domain 11 (green triangles) is shown in Fig. 1B.

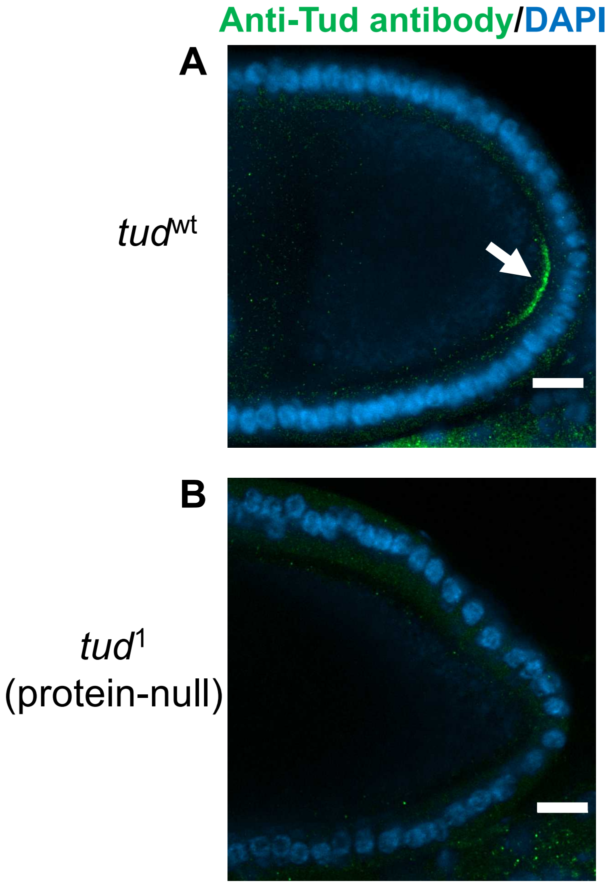

Figure S2. Validation of rabbit anti-Tudor antibody.
(A) Tud protein in the germ plasm at the posterior pole of the wild-type oocyte (tud^{wt}) at a mid-oogenesis stage is labeled with new rabbit anti-Tud antibody (green, arrow, Materials and Methods). Nuclei of follicle cells surrounding the oocyte are labeled with DAPI. For these immunostaining experiments, ovaries from tud^{1}/*CyO*, which carry one copy of *wt tud* allele on *CyO* balancer chromosome along with the original protein-null tud^{1} mutant allele (E. Wieschaus and C. Nüsslein-Volhard, unpublished; sequenced in (Arkov AL et al, 2006)), were used. **(B)** Specificity of the antibody was further confirmed in immunostaining experiments using ovaries from females transheterozygous for tud^{1} and *tud* deletion $Df(2R)Pu^{rP133}$. As expected, no Tud was detected in the germ plasm of the oocytes in the tud^{1} mutant ovaries. In addition, this antibody was validated in western-blot experiments (Fig. 4). Scale bars are 15 μm.

References

Arkov AL, Wang JY, Ramos A, Lehmann R (2006) The role of tudor domains in germline development and polar granule architecture. *Development (Cambridge, England)* 133: 4053-4062.

Boswell RE, Mahowald AP (1985) Tudor, a gene required for assembly of the germ plasm in drosophila melanogaster. *Cell* 43: 97-104. doi:10.1016/0092-8674(85)90015-7

Liu H, Wang JY, Huang Y, Li Z, Gong W, Lehmann R, Xu RM (2010) Structural basis for methylarginine-dependent recognition of aubergine by tudor. *Genes Dev* 24: 1876-1881.
